# Evaluation of the efficacy of 1 L polyethylene glycol plus ascorbic acid and an oral sodium sulfate solution: A multi-center, prospective randomized controlled trial

**DOI:** 10.1097/MD.0000000000030355

**Published:** 2022-09-02

**Authors:** Jung Hun Woo, Hoon Sup Koo, Dae Sung Kim, Jeong Eun Shin, Yunho Jung, Kyu Chan Huh

**Affiliations:** a Department of Internal Medicine, Konyang University College of Medicine, Daejeon, Republic of Korea; b Department of Internal Medicine, Dankook University College of Medicine, Cheonan, Republic of Korea; c Department of Internal Medicine, Soonchunhyang University College of Medicine, Cheonan, Republic of Korea.

**Keywords:** cathartics, colonoscopy, polyethylene glycols, sodium sulfate

## Abstract

Low-volume bowel preparation has been developed to increase patient compliance. We compared 1 L of polyethylene glycol/ascorbic acid (PEG/Asc) and oral sodium sulfate (OSS) with respect to bowel preparation efficacy, compliance, and safety.

**Methods:**

A multicenter, prospective, randomized, single-blinded, non-inferiority trial was conducted in 3 hospitals. Patients were randomized to receive a bowel-cleansing agent. Bowel-cleansing efficacy was evaluated using the Boston Bowel Preparation Scale (BBPS). Satisfaction, feeling, taste of the bowel cleanser, and adverse events after taking the bowel cleanser were investigated through a questionnaire. Additionally, blood samples were analyzed before and after bowel cleansing.

**Results:**

In total, 172 patients were analyzed (85 with 1 L PEG/Asc and 87 with OSS), and the mean BBPS scores were comparable between agents. The 1L PEG/Asc group tended to have a higher BBPS score in the right colon (2.22 vs 2.02; *P* = .08). The compliance of 1 L of PEG/Asc was comparable to that of OSS. Patients taking 1 L PEG/Asc reported greater thirst and dizziness (*P* = .04 and *P* = .047, respectively) than the OSS cohort. On the other hand, gastrointestinal symptoms such as vomiting and abdominal distension were more common in the OSS group, without statistical significance. In terms of laboratory adverse events, elevation of serum creatinine was found in both groups after taking the bowel cleansing agent (*P* < .001 for the 1L PEG/Asc group; *P* = .04 for the OSS group). However, most of the increased values were within the normal ranges.

**Discussion:**

The 1L PEG/Asc treatment was comparable to OSS in terms of bowel preparation efficacy, compliance, and safety.

## 1. Introduction

Colorectal cancer (CRC) is the third most common cancer worldwide with regard to frequency and mortality^[1]^ and is accurately diagnosed through colonoscopic examination.^[2]^ High-quality colonoscopy can reduce the CRC mortality rate by 53% and incidence by 76%.^[3]^ Data regarding the adenoma detection rate (ADR), adenoma miss rate, cecal intubation rate, and bowel preparation are required for high-quality colonoscopy.^[4]^ Bowel preparation is important because optimal bowel preparation determines the accuracy, speed, and rates of successful cecal intubation during colonoscopy. Incomplete bowel preparation is associated with a 3-fold reduction in ADR, high complication rates, and the additional costs of a repeat procedure.^[5–9]^ an optimal bowel preparation method improves patient compliance and minimizes adverse events, such as electrolyte imbalance and intestinal tract injury.^[10]^

The most commonly used bowel cleansers are classified into the following 3 categories: polyethylene glycol (PEG), osmotic laxatives, and irritant laxatives.^[11]^ Among these, 4 L polyethylene glycol electrolyte lavage solution (PEG-ELS) has been safely and widely used since the 1990s. Sulfate ions in PEG-ELS replace chloride ions in the intestine and reduce the active absorption of sodium, with consequent diarrhea and without electrolyte imbalance or intestinal tract injury.^[12]^ Despite these advantages, 4 L PEG-ELS is poorly tolerated owing to the volume of solution that needs to be consumed and its salty taste. Therefore, low-volume bowel cleansers have been introduced recently to overcome the disadvantages of the conventional PEG formulation.^[13]^ Low volume bowel cleansers include 2 L PEG/ascorbic acid (PEG/Asc), 1 L PEG/Asc, oral sodium sulfate (OSS), oral sodium phosphate, and sodium picosulfate with magnesium citrate (SPMC).

The 2 L PEG/Asc preparation contains a mixture of PEG and ascorbic acid. Ascorbic acid promotes osmosis, improves taste, and reduces the volume of the solution from 4 L to 2 L.^[14]^ A previous study reported that the efficacy of 2 L PEG/Asc was comparable with that of 4 L PEG, with better patient compliance.^[15]^ CleanViewAL (Taejoon Pharm, Seoul, Korea) is a 1 L PEG/Asc formulation that was recently introduced in Korea and contains 160 g of PEG, 18 g of anhydrous sodium sulfate, 2.7 g of sodium chloride, 1 g of potassium chloride, 40.6 g of Asc, and 9.4 g of sodium ascorbate. The ascorbic acid ratio is higher in 1 L PEG/Asc than in 2 L PEG/Asc. A meta-analysis reported that split-dose 1 L PEG/Asc had a higher right polyp detection rate and better bowel-cleansing efficacy than split-dose 2 L PEG/Asc.^[16]^

OSS is an osmotic laxative that is not absorbed by the intestine and is used in Korea as a SUPREP bowel cleanser (Taejoon Pharm, Seoul, Korea). SUPREP contains 35 g of anhydrous sodium sulfate, 6.26 g of potassium sulfate, and 3.2 g of anhydrous magnesium sulfate. A previous study has shown that the rate of successful bowel preparation was higher in the OSS group than in the 4 L sulfate-free ELS group, with comparable safety profiles for both preparations.^[17]^ Another study reported that OSS and low-volume PEG were similar in terms of patient satisfaction; however, mild gastrointestinal adverse effects, such as nausea and abdominal pain, were relatively common.^[18]^

Several studies have investigated methods to reduce the volume and enhance the taste of bowel cleanser preparations, to improve patient compliance and reduce adverse effects. Many low-volume bowel cleansers have been developed, and a few studies have compared low-volume bowel cleansers. However, a limited number of studies have described the use of 1 L PEG/Asc to enhance the taste of bowel cleansers and improve patient compliance. In this study, we compared OSS, which has been previously investigated, and 1 L PEG/Asc with regard to bowel preparation efficacy, compliance, and safety.

## 2. Methods

### 2.1. Study design

This multicenter, prospective, randomized, single-blinded, non-inferiority trial was conducted at 3 hospitals in South Korea. The study received institutional review board approval (no. 2019-05-020) and was conducted in accordance with the principles of the Declaration of Helsinki and Good Clinical Practice. The endoscopists who conducted the research were faculty members at each university hospital. All patients were informed of the study and provided informed consent before participation. After obtaining informed consent, the patients were randomly assigned to 2 groups: the 1 L PEG/Asc (CleanViewAL, Taejoon Pharm, Seoul, Korea) and OSS (SUPREP, Taejoon Pharm, Seoul, Korea).

### 2.2. Subjects

The subjects were enrolled from August 2020 to January 2021. Outpatients aged 20 to 65 years who underwent colonoscopy for diagnosis, screening, surveillance, or treatment were included. Patients with disabilities (cognitive disorders, intellectual disabilities), intestinal obstruction, severe constipation (defined as patients who has less than 3 bowel movements per week or who used laxatives), history of intestinal surgery, ascites, heart failure, heart disease (ischemic heart disease, coronary artery disease within the last 6 months), inflammatory bowel disease, pregnancy, kidney failure (serum creatinine >3 mg/dL for more than 6 months), electrolyte abnormalities observed prior to taking bowel cleansers, and those who refused consent were excluded.

### 2.3. Bowel preparation

The patients were randomly assigned to receive 1 L of PEG/Asc and OSS. Patients in both groups were recommended a low-fiber diet for 3 days before colonoscopy and were only allowed to eat a liquid diet for dinner on the day before the examination. The regimen for bowel cleansers was a split-dosing regimen that indicated for the patients to take bowel cleansers the day before and the day of colonoscopy.

In the 1 L PEG/Asc group, half dose of PEG/Asc solution (500 mL) was taken for 30 minutes, and then, 500 mL of water was taken for 30 minutes at 8:00 pm on the day before colonoscopy. Then, 4 to 6 hours before the examination, the remaining half dose of PEG/Asc solution (500 mL) and 500 mL of water were taken in the same way. If the total amount of PEG/Asc was not fully removed, the remaining volume was noted. In addition, if the patient consumed additional water, the water volume was noted.

In the OSS group, to prepare the preparation solution, water was added to 177 mL of OSS solution for a total volume of 480 mL. The day before colonoscopy, the OSS solution was administered as described above, and then, the subjects drank 480 mL of water twice more over 1 hour. On the day of colonoscopy, 4 to 6 hours before the examination, the patient group took OSS preparations and water in the same way as before. Similar to the previous group, the remaining amount of OSS solution and the amount of additional water intake were measured.

### 2.4. Assessments

1. Bowel cleansing efficacy

Bowel-cleansing efficacy was scored by a blinded endoscopist who performed colonoscopy using the Boston Bowel Preparation Scale (BBPS). BBPS scores are widely used to determine the quality of colon cleansing and range from 0 to 3 points.^[19]^ Each BBPS score is defined as follows; 0 = unprepared colon segment with mucosa invisible because of remnants of solid stool; 1 = part of the mucosa of the colon segment can be seen but the other colon cannot be seen due to residual stool, opaque liquid; 2 = most mucosa of the colon segment can be seen with residual staining, small stool fragments; 3 = Entire mucosa of the colon segment can be seen without any remnant, fragments.

The colon was divided into 3 sections: the right colon, from the cecum to the ascending colon, the transverse colon, from the hepatic flexure to the splenic flexure, and the left colon, from descending colon to rectum. Each segment was assigned a BBPS. Bowel cleansing was considered “appropriate” when the BBPS score was >2 in each section (≥6). Cleansing was considered “inappropriate” if any section scored one or less, or if the total score was 5 or less.

1. Compliance

Patient compliance was assessed using a questionnaire before colonoscopy. The questions included whether bowel cleansers were taken completely, all accompanying water was taken, and water was taken in addition to the prescribed amount. Furthermore, the questionnaire asked about the feeling and taste of the bowel cleansers (5 points: very satisfied; 4 points: satisfied; 3 points: neutral, 2 points: dissatisfied; 1: very dissatisfied; 5-point Likert scale) and satisfaction with bowel-cleansing preparations (0: very dissatisfied; 10: very satisfied; visual analog scale [VAS]).

1. Safety

Safety was evaluated from 2 perspectives: clinical and laboratory. Clinical safety was confirmed through a questionnaire about gastrointestinal symptoms (nausea, vomiting, abdominal pain, abdominal distension, and thirst) and neurological symptoms (dizziness, numbness, and paresthesia). Laboratory safety was confirmed by blood tests. A baseline blood test was performed, and a blood test was repeated on the day of the procedure to determine whether abnormalities occurred. The blood tests included blood urea nitrogen, creatinine, sodium, potassium, and chloride level measurements.

### 2.5. Statistical analysis

Continuous variables such as BBPS, Likert scale, and VAS score were analyzed using a Student *t* test or Mann–Whitney *U* test, as appropriate. Categorical variables, such as sex, indications for colonoscopy, adverse events, and adequacy of bowel preparation, were compared using the Pearson chi-square test or Fisher exact test, as appropriate. The statistical significance of changes in blood test results before and after the procedure for each group was identified using the paired *t* test or Wilcoxon signed rank test, as appropriate. All analyses were performed using SPSS software (version 20.0; IBM Corp., Armonk, NY), and *P* values <.05 were considered statistically significant.

## 3. Results

### 3.1. Patients

A total of 180 patients were enrolled and randomized into the same ratio (90 1 L PEG/Asc, 90 OSS). Among them, 8 patients were excluded from the study due to withdrawal of consent (n = 2) and follow-up loss (n = 6). Therefore, the analysis was performed on 172 patients (85 1 L PEG/Asc, 87 OSS). Demographic characteristics such as age, sex, and indications for colonoscopy are shown in Table 1. There were no significant differences between the 2 groups.

**Table 1:** Demographic characteristics of the study population.

	1 L PEG/Asc (n = 85)	OSS (n = 87)	*P* value
Age			
Median (range), yr	56 (20–71)	57 (25–71)	.32
20–64, n (%)	77 (90.6)	71 (81.6)	.09
≥65, n (%)	8 (9.4)	16 (18.4)	
Sex, n (%)			.21
Male	53 (62.4)	46 (52.9)	
Indications for colonoscopy, n (%)			.25
Screening	5 (5.9)	10 (11.5)	
Surveillance	32 (37.6)	25 (28.7)	
Diagnostics	26 (30.6)	22 (25.3)	
Treatment	22 (25.9)	30 (34.5)	

OSS = oral sodium sulfate, PEG/Asc = polyethylene glycol/ascorbic acid.

### 3.2. Bowel preparation efficacy

There were no differences in the adequacy of bowel preparation, with 98.8% (84/85) and 96.6% (84/87) of individuals in the 1 L PEG/Asc and OSS groups achieving adequate preparations, respectively. The 1 L PEG/Asc group tended to have a higher BBPS score for the right colon than the OSS group (2.22 vs 2.02; *P* = .08), but the other colon segments, such as the transverse colon, left colon, and overall colon, tended to have lower BBPS scores in this group. Notably, no significant differences were observed between the groups (Table 2).

**Table 2:** The adequacy of the bowel preparation according to the type of method.

	1 L PEG/Asc (n = 85)	OSS (n = 87)	*P* value
BBPS, mean (SD)			
Right colon	2.22 (0.68)	2.02 (0.75)	.08
Transverse colon	2.05 (0.74)	2.11 (0.83))	.52
Left colon	2.21 (0.77)	2.23 (0.90)	.67
Overall colon	7.66 (1.18)	7.84 (1.39)	.13
Adequacy, n (%)			
Adequate	84 (98.8)	84 (96.6)	.62
Inadequate	1 (1.2)	3 (3.4)	

BBPS = Boston Bowel Preparation Scale, OSS = oral sodium sulfate, PEG/Asc = polyethylene glycol/ascorbic acid, SD = standard deviation.

**Table 3:** Completion of the bowel preparation protocol.

	1 L PEG/Asc (n = 85)	OSS (n = 87)	*P* value
Bowel cleanser intake			.50
Complete, n (%)	85 (100.0)	85 (97.7)	
Water intake			>.99
Complete, n (%)	82 (96.4)	84 (96.6)	

OSS = oral sodium sulfate, PEG/Asc = polyethylene glycol/ascorbic acid.

### 3.3. Compliance

There were no significant differences between the 1 L PEG/Asc and OSS groups in the completion of bowel cleaning (100% vs 97.7%; *P* = .50) and of water intake (96.4% vs 96.6%; *P* > .99; Table 2). In 1 L PEG/Asc group, the average Likert score for feeling (3.52 vs 3.47; *P* = .94) and taste (2.92 vs 2.70; *P* = .21) and the VAS score for satisfaction (7.06 vs 7.01; *P* = .68) tended to have higher values than those of the OSS group, without statistical significance (Table 4).

**Table 4:** Patient satisfaction with bowel cleansing agents.

	1 L PEG/Asc (n = 85)	OSS (n = 87)	*P* value
Feeling, mean (SD)*	3.52 (0.97)	3.47 (0.98)	.94
Taste, mean (SD)*	2.92 (0.83)	2.70 (0.84)	.21
Satisfaction, mean (SD)†	7.06 (1.96)	7.01 (2.45)	.68

OSS = oral sodium sulfate, PEG/Asc = polyethylene glycol/ascorbic acid, SD = standard deviation.

* Likert scale.

† Visual analog scale (VAS).

### 3.4. Safety

1. Clinical safety

The frequencies of adverse event are shown in Table 5. The 1 L PEG/Asc group reported thirst (30.6% vs 17.2%; *P* = .04) and dizziness (25.9% vs 13.8%; *P* = .047) to a greater extent than the OSS group. Other adverse events included nausea (42.4% vs 44.8%; *P* = .74), vomiting (10.6% vs 12.6%; *P* = .67), abdominal pain (18.8% vs 17.2%; *P* = .79), and numbness in the hands and feet (2.4% vs 2.3%; *P* > .99), and there were no statistically significant differences.

**Table 5:** Adverse events in bowel preparations.

	1 L PEG/Asc (n = 85)	OSS (n = 87)	*P* value
Symptoms, n (%)			
Nausea	36 (42.4)	39 (44.8)	.74
Vomiting	9 (10.6)	11 (12.6)	.67
Abdominal pain	16 (18.8)	15 (17.2)	.79
Abdominal distension	31 (36.5)	36 (41.4)	.51
Thirst	26 (30.6)	15 (17.2)	.04
Dizziness	22 (25.9)	12 (13.8)	.047
Paresthesia	0 (0.0)	1 (1.1)	>.99
Numbness in hands and feet	2 (2.4)	2 (2.3)	>.99

OSS = oral sodium sulfate, PEG/Asc = polyethylene glycol/ascorbic acid.

1. Laboratory safety

The laboratory profiles of the 1 L PEG/Asc group and OSS groups are shown in Table 6. In the 1 L PEG/Asc group, the median serum creatinine level (baseline 0.77 [range, 0.51–1.59]; exam day 0.91 [range, 0.53–1.88]; *P* < .001), median potassium level (baseline 4.3 [range, 4.0–5.0]; exam day 4.6 [range, 3.4–5.8]; *P* < .001), and median chloride level (baseline, 104 [range, 96–111]; exam day, 108 [range, 101–116]; *P* < .001) significantly increased after taking a bowel cleanser. Most of the increased values after bowel preparation were within the reference range. Similarly, in OSS group, the median creatinine level elevated after taking bowel cleanser (baseline 0.78 [range, 0.49–1.19]; exam day 0.78 [range, 0.49–1.2]; *P* = .04) but remained within a normal range.

**Table 6:** Laboratory profiles before and after bowel cleanser administration.

	1 L PEG/Asc (n = 85)			OSS (n = 87)		
	Baseline	Exam day	*P*	Baseline	Exam day	*P*
BUN, mg/dL, median (range)	13.7 (6.9–24.6)	13.9 (5.7–24.8)	.60	13.3 (6.3–25.6)	12.6 (6.4–24.5)	.005
Creatinine, mg/dL, median (range)	0.77 (0.51–1.59)	0.91 (0.53–1.88)	<.001	0.78 (0.49–1.19)	0.78 (0.49–1.2)	.04
Sodium, mmol/L, median (range)	141 (133–145)	140 (134–147)	.84	140 (135–146)	140 (133–145)	.04
Potassium, mmol/L, median (range)	4.3 (4.0–5.0)	4.6 (3.4–5.8)	<.001	4.3 (4.0–5.0)	4.3 (3.7–5.1)	.75
Chloride, mmol/L, median (range)	104 (96–111)	108 (101–116)	<.001	104 (94–105)	103 (95–107)	.93

BUN = blood urea nitrogen, OSS = oral sodium sulfate, PEG/Asc = polyethylene glycol/ascorbic acid.

## 4. Discussion

Colonoscopy is considered the most sensitive method for diagnosing CRC, and optimal bowel preparation is important for this purpose. Conventionally, 4 L PEG is the most common bowel cleanser used in clinical practice; however, the large volume and salty taste of this solution are associated with poor tolerability. Furthermore, 5% to 15% of patients do not adequately comply with the complete bowel cleansing regimen.^[20]^ Recent studies have described a low-volume bowel cleanser that scores over conventional bowel cleansers with regard to taste and volume of the preparation, with comparable safety and efficacy. In this study, we compared the efficacy, compliance, and safety of 1 L of PEG/Asc and OSS.

A prior study that compared 1 L PEG/Asc and SPMC reported non-inferiority of bowel preparation in the 1 L PEG/Asc group. Based on the Harefield cleansing scale (HCS), the high-quality bowel cleansing rate of 1 L PEG/Asc versus SPMC in the right colon was 4.4% versus 1.2% (*P* = .03).^[21]^ In a recent study that compared 1 L PEG/Asc and 2 L PEG/Asc with regard to colon cleansing efficacy and safety, 1 L PEG/Asc administered as an evening/morning split dose or as a morning-only regimen was compared to 2 L PEG/Asc. Based on the HCS scores, high-quality bowel preparation of the right colon was statistically superior to that observed with 2 L PEG/Asc in the 1 L PEG/Asc evening/morning split-dose group (*P* = .01).^[16]^ Recent research has also highlighted the importance of thorough bowel cleansing of the right colon during colonoscopy. Flat lesions and serrated adenomas are more common in the right than in the left colon.^[19]^ Endoscopic detection of such lesions is challenging, and the lesions can be missed, resulting in a high incidence of interval cancer. Therefore, meticulous right colonic bowel cleansing is essential for preventing interval cancer. In our study, we observed no difference between the 1 L PEG/Asc and OSS groups with regard to bowel cleansing efficacy throughout the colon. The BBPS scores for right colon cleansing were higher in the 1 L PEG/Asc than in the OSS group (2.22 vs 2.02; *P* = .07), which, although statistically insignificant, is a meaningful result. The selection of a bowel preparation that is associated with high BBPS scores for right colon cleansing may be a useful preventive strategy against missing neoplastic lesions.

The VAS scores for feeling and taste and the Likert satisfaction scores were higher in the 1 L PEG/Asc than in the OSS group, which suggests that the possibility of using the same product for the next colonoscopy is high. However, the difference was not statistically significant. Safety was evaluated based on clinical and laboratory safety, and clinical safety was determined based on gastrointestinal and neurological symptoms. Gastrointestinal symptoms such as nausea, vomiting, and abdominal distention were more common in the OSS group than in the 1 L PEG/Asc group, which may be attributed to the fact that the volume of bowel cleanser administered to the OSS group was larger than that administered to the 1 L PEG/Asc group; however, this difference was not statistically significant. Notably, patients who received 1 L of PEG/Asc reported greater thirst than those who received OSS (30.6% vs 17.2%; *P* = .04). Furthermore, we observed no significant intergroup clinical differences in the neurological symptoms. However, dizziness was more common in the 1 L PEG/Asc than in the OSS group (25.9% vs 13.8%; *P* = .047). Sodium ascorbate and sodium sulfate in the 1 L PEG/Asc formulation produced dehydration, which resulted in increased thirst and dizziness. Therefore, sufficient fluid intake is important to prevent dehydration in patients who receive this preparation.

With regard to laboratory safety, both groups showed elevated serum creatinine levels after bowel cleanser administration. However, the elevated levels usually remained within the reference range and did not produce clinically significant effects in healthy adults. Nevertheless, these results are important and may serve as guidelines for the selection of a bowel cleanser for patients with chronic kidney disease, elderly patients, and those with heart failure.

In summary, 1 L PEG/Asc and OSS are comparable with regard to efficacy, safety, and patient compliance. Our multi-center randomized prospective controlled trial is the first Korean study and the first study in an Asian population to evaluate 1 L PEG/Asc as a bowel cleanser. The small sample size (patient group, as well as the center at which the study was performed) is a limitation of the study; therefore, most results were not statistically significant. Large-scale, multicenter studies are warranted to confirm our findings.

## Author contributions

Study concept and design: KCH. Data acquisition: JHW, HSK, DSK, JES, YJ, and KCH. Data analysis and interpretation: JHW, HSK, DSK, JES, YJ, KCH, and drafting of the manuscript: JHW and HSK. Critical revision of the manuscript for important intellectual content: HSK and KCH. Obtained funding: KCH. Approval of final manuscript: all authors.

Conceptualization: Kyu Chan Huh.

Data curation: Dae Sung Kim, Hoon Sup Koo, Jeong Eun Shin, Jung Hun Woo, Kyu Chan Huh, Yunho Jung.

Formal analysis: Dae Sung Kim, Hoon Sup Koo, Jeong Eun Shin, Jung Hun Woo, Kyu Chan Huh, Yunho Jung.

Funding acquisition: Kyu Chan Huh

Supervision: Kyu Chan Huh.

Writing – original draft: Hoon Sup Koo, Jung Hun Woo.

Writing – review & editing: Kyu Chan Huh.
